# Military Nursing Services of the Crown

**Published:** 1918-11-09

**Authors:** 


### MILITARY AND OVERSEAS MATRONS-IN-CHIEF.

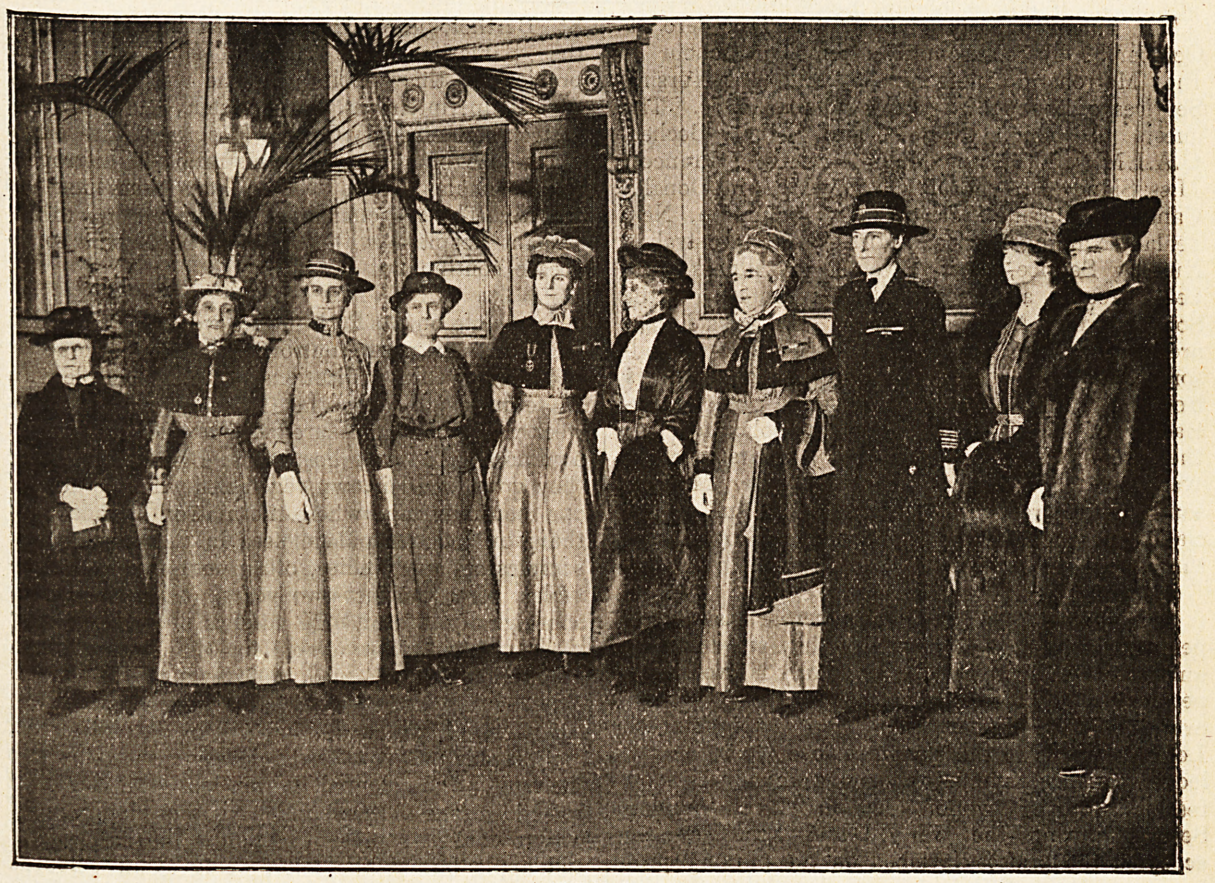

Miss Swift; Miss Thurston (New Zealand); Dame Maud McCarthy (France); Miss Macdonald (Canada); Dame Ethel Becher Q.A.I.M.N.S.; H.R.H. Princess Louise, Duchess of Argyll; Miss Sidney Browne, T.F.N.S.; Lady Ampthill, V.A.D.; Lady Lawrence, Welfare Dept. Ministry of Munitions; Mrs. Massey Lyon, Chairman of Committee

### Military Nursing Services of the Crown.

THE above excellent group of the Matrons-in-Chief of the Military Hospitals, which also includes the Matron-in-Chief of the British Red Cross Society, Miss Swift, R.R.C., will possess a permanent interest and should prove historical. Every one who is in any way connected with the administration of an efficient hospital will not fail to appreciate the immense output of energy, personal effort, and devotion represented by the forces controlled by the Matrons-in-Chief, with Lady Ampthill, whose portraits are included in the above group. They extend to the provision made for wounded and injured warriors engaged in the greatest war the world has ever witnessed, embracing from seven to nine separate fronts and campaigns all proceeding at one and the same time. The Joint War Committee's field of work, for which Miss Swift is responsible as Matron-in-Chief, included Red Cross and Auxiliary Hospitals, a variety of other hospitals situated in France, Belgium, and various other countries. There were also nurses' departments in connection with Home Hospitals, where 2,393 additional nurses were posted during the year ending 1917, and 372 nurses to foreign service.

In October, 1914, the Red Cross Service included the following trained nurses, 364 St. John's nurses and 230 Red Cross nurses, posted to hospitals abroad, including French hospitals where French patients were nursed. In addition to these there were 300 British Red Cross and St. John's nurses engaged on Home service. The first batch of nurses of the Order of St. John (the two organisations not then working together) were sent out on 15th August, 1914, thirty of them to Brussels. At the present time 2,000 Joint Committee trained nurses are engaged on Home service and 500 on foreign service. There are some thousands of V.A.D.s engaged on foreign service under the Joint Committee, of which the precise numbers are not forthcoming, but they probably amount to, if they do not exceed, the number employed by Queen Alexandra's Imperial Military Nursing Service and its Reserves, whom Dame Ethel Becher put at 6,000, "without whose help it would have been impossible to have carried on the work of the military hospitals." Dame Becher would like "to express to the Matrons-in-Chief of the Dominions her personal gratitude for the help and co-operation received from them whenever their work had brought them in contact." The figures here given are exclusive of the nurses and V.A.D.s working under the Territorial Force Nursing Service, the numbers of which we hope to be in a position to publish next week.

Our available space does not permit us to publish a verbatim report of the interesting speeches and proceedings at the Luncheon given to the Military and Overseas Matrons-in-Chief. The best report will be found in the *Daily Telegraph* for October 31, page 9, and we can confidently recommend all of our readers to obtain a copy of that paper for permanent preservation and record.

It remains to express the warmest admiration for the spontaneous and successful organisation of the Luncheon given in honour of the Matrons-in-Chief of the Military Services of the Crown as a mark of appreciation of the noble work performed by the nursing profession during the war. The moving spirit was Mrs. Massey Lyon, Chairman, Lady Editor of the *Queen*, which we can testify from fulness of knowledge has rendered for many years yeoman service in support of every good movement for the social welfare of the people, and has done much to further these objects. The *Queen* is a great paper, and Mrs. Massey Lyon was a tower of strength, and proved herself an ideal Chairman. The idea of this memorable Luncheon originated with Miss M. F. Billington, President of the Society of Women Journalists, who acted as Honorary Secretary. The thanks of the nation are especially due to Mrs. Massey Lyon and Miss F. M. Billington, whose splendid organising powers and influence were never better employed. They have won for themselves the grateful recognition and thanks of all classes throughout the nation.

